# Anatomical inputs to sulcal portions of areas 9m and 8Bm in the macaque monkey

**DOI:** 10.3389/fnana.2015.00030

**Published:** 2015-03-12

**Authors:** Manoj K. Eradath, Hiroshi Abe, Madoka Matsumoto, Kenji Matsumoto, Keiji Tanaka, Noritaka Ichinohe

**Affiliations:** ^1^Laboratory for Cognitive Brain Mapping, RIKEN Brain Science InstituteSaitama, Japan; ^2^Graduate School for Science and Engineering, Saitama UniversitySaitama, Japan; ^3^Ichinohe Neural System Group, Laboratory for Molecular Analysis of Higher Cognitive Function, RIKEN Brain Science InstituteSaitama, Japan; ^4^Department of Neuropsychiatry, The University of Tokyo HospitalBunkyo, Japan; ^5^Brain Science Institute, Tamagawa UniversityMachida, Japan; ^6^Department of Ultrastructural Research, National Centre of Neurology and Psychiatry, National Institute of NeuroscienceKodaira, Japan

**Keywords:** retrograde tracer, labeled cell bodies, connections, area 9m, area 8Bm

## Abstract

Neuronal activities recorded from the dorsal bank of the anterior cingulate sulcus have suggested that this cortical area is involved in control of search vs. repetition, goal-based action selection and encoding of prediction error regarding action value. In this study, to explore potential anatomical bases for these neuronal activities, we injected retrograde tracers (CTB-Alexa-488 and CTB-gold) into the dorsal bank of the anterior cingulate sulcus and examined the distribution of labeled cell bodies in macaque monkey brains. The Nissl staining showed that the cortex in the dorsal bank of the anterior cingulate sulcus has consistent layer 4 which means that the cortical region is a part of the granular prefrontal cortex. The injection site belonged to the sulcal portion of area 9m in two cases and the sulcal portion of area 8Bm in one case. In addition to the continuous distribution of labeled cells in the two areas (areas 9m and 8Bm) around the injection site, the labeled cells were densely distributed in the cingulate areas (areas 24, 32, and 23) in all the cases. The dense labeling of cells was also found in other prefrontal areas (areas 46, 10, 11, and 12) in the two cases with injection into the sulcal portion of area 9m, whereas the dense labeling of cells was found in pre-motor areas (F6 and F7) in the case with injection into the sulcal portion of area 8Bm. The dense labeling of cells in the prefrontal and premotor areas was more similar to those previously found after injections into dorsal parts of areas 9 and 8B. Subcortical distribution of labeled cells was found in the mediodorsal nucleus of thalamus, claustrum, and substantia nigra pars compacta in all the cases.

## Introduction

Amongthe medial prefrontal and frontal cortical areas, the dorsal bank of the anterior cingulate sulcus has drawn special interests, as several distinguishing neuronal activities have been recorded from this cortical area in macaque monkeys. Electrophysiological recordings from behaving macaque monkeys showed that activities of neurons in the area represent action sequences specifically during search or during repetition of correct sequences (Procyk et al., 2000), action-outcome contingencies during learning (Matsumoto et al., 2003; Cai and Padoa-Schioppa, 2012), both positive and negative prediction errors regarding action values (Matsumoto et al., 2007; Quilodran et al., 2008) and history of erroneous responses (Kuwabara et al., 2014). These diverse neuronal signals suggest that the goal directed action plans are integrated with outcome evaluations in the dorsal bank area of anterior cingulate sulcus. The cortex in the dorsal bank of the anterior cingulate sulcus in macaque brain has layer 4 although less distinguished than that in the lateral prefrontal areas (Barbas and Pandya, 1989; Petrides and Pandya, 1999), which means that it belongs to the granular prefrontal cortex. The neighboring cortex in the ventral bank of the anterior cingulate sulcus lacks layer 4 and thus belongs to the agranular cingulate cortex: it has been labeled as area 24c (Matelli et al., 1991; Arikuni et al., 1994). Whether the pattern of anatomical inputs to the dorsal bank of the anterior cingulate sulcus is similar to that to the ventral neighbor (a part of the cingulate cortex) or the dorsal neighbor (a part of the prefrontal cortex) is of special interests. Although there are several tracing studies using macaque monkeys in which retrograde tracers were injected into the medial surface of anterior cingulate cortex ventral to the anterior cingulate sulcus (Barbas and Pandya, 1989; Arikuni et al., 1994; Carmichael and Price, 1995a,b, 1996) or into the dorsal parts of areas 9m and 8Bm on the medial surface above the anterior cingulate sulcus (Petrides and Pandya, 1999; Saleem et al., 2014), there were no cases in which retrograde tracers were injected into the dorsal bank of the anterior cingulate sulcus. This lack of anatomical studies makes it difficult to discuss the anatomical bases of the neuronal activities reported from the dorsal bank of the anterior cingulate sulcus. As the mediodorsal parts of the prefrontal cortex have been distinguished as area 9m (anteriorly) and area 8Bm (posteriorly) (Walker, 1940; Petrides and Pandya, 1999), the dorsal bank of the anterior cingulate sulcus should be the most ventral parts of areas 9m and 8Bm. To examine anatomical inputs to the dorsal bank of the anterior cingulate sulcus, we injected retrograde tracers (CTB-Alexa-488 and CTB-gold) into the sulcal portions of areas 9m and 8Bm and examined the distribution of labeled cell bodies in cortical and subcortical structures in macaque monkeys.

## Materials and methods

We used two male rhesus monkeys (*Macaca mulatta*) weighing 7–10 kg. The two monkeys (Monkey 1 and Monkey 2) used in this study were the same animals used in our electrophysiological studies (Matsumoto et al., 2006, 2007). We kept the animal number codes consistent between this study and the previous studies. All procedures were approved by the RIKEN Animal Experiment Committee and were in accordance with the Guideline for Animal Experiments of the Japan Neuroscience Society.

The surgical procedures, task, training, and electrophysiological experiments have already been described with electrophysiological results (Matsumoto et al., 2006, 2007). After confirming the location of the targeted part of the dorsal bank of the anterior cingulate sulcus by anatomical MRI, a head holder and two electrophysiology recording chambers (20 mm in diameter) were implanted by an aseptic surgery under phenobarbital–induced anesthesia (35 mg per kilogram body weight, intraperitoneal injection).

**Figure 1:**
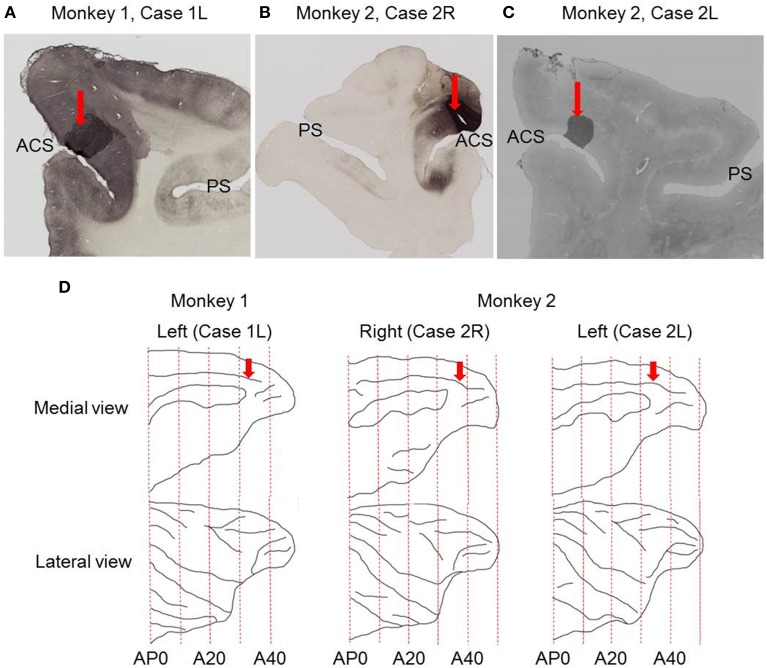
**Sites of tracer injections. (A)** Photomicrograph of coronal section showing the site of CTB-Alexa-488 injection in Case 1L. **(B)** Photomicrograph of coronal section showing the site of CTB-Alexa-488 injection in Case 2R. **(C)** Photomicrograph of coronal section showing the site of CTB-gold injection site in Case 2L. **(D)** Medial and lateral views of the brain reconstructed from structural MRI images. Red arrows indicate the sites of tracer injections into the dorsal bank of the anterior cingulate sulcus. ACS, anterior cingulate sulcus; PS, principal sulcus.

Retrograde tracers were injected into the dorsal bank of the anterior cingulate sulcus (Figure 1). During tracer injections, the animals were fully awake with the head fixed in the monkey chair. A 24G stainless steel needle was filled with retrograde tracers, CTB-Alexa-488 (Invitrogen-Molecular Probes, Eugene, OR) or 7-nm colloidal gold (CTB-gold, List Biological Laboratories, Inc., Campbell, CA), diluted in 0.1 M phosphate-buffered saline (PBS). The needle filled with the tracers was connected to a silicon tube filled with PBS and further connected to a 10 μl micro syringe filled with PBS (Hamilton, Reno, NV). The needle was attached to an oil hydraulic micromanipulator (Narishige, Japan) and slowly advanced through the same grid as that used for single-cell recordings to the dorsal bank of the anterior cingulate sulcus. The depth location of the anterior cingulate sulcus was determined during recordings with an electrode by characteristic absence of neuronal activities in the sulcus. The needle tip was first advanced to 0.52 mm above the dorsal surface of the anterior cingulate sulcus, and then withdrawn to 1.00 mm above the dorsal surface of the anterior cingulate sulcus to make a space for the tracers to stay in the gray matter. We left the needle at that position for about 5 min and then 1 μl of tracers were injected over a period of 10 min using a syringe pump (KDS210, KD Scientific Inc. Holiston, MA). The needle was left at that position for another 5 min and then slowly retracted.

The animals were kept in their home cages for 1 week for the tracer transport after the tracer injections, and then they were perfused first with 2 L of phosphate buffer (pH 7.3), followed by 4 L of a solution containing 4% paraformaldehyde in PB, 20% sucrose in PB, and 30% sucrose in PBS. The brain was subsequently removed from the skull and cut sagittally. After removing the pons and cerebellum, the brain specimens were kept in 30% sucrose in PB. After the brain specimens sank, we cut them into 50 μm-thick sections with a sliding microtome. The sections were divided into five series. The first series were used for visualizing either Alexa488 or Gold. The second series were stained for the Nissl substance with thionin. The contralateral hemispheres were not stained for the injected tracers.

## Immuno-histochemical staining for Alexa-488

Sections were incubated for 1 h with 0.1 M PBS (pH 7.3), containing 0.5% Triton X-100 and 5% normal goat serum (PBS-TG) at room temperature and then for 40–48 h at 4°C with PBS-TG containing a monoclonal anti-Alexa-488 antibody (Invitrogen-Molecular Probes, Eugene, OR, 1:1000). After rinsing, the sections were placed in PBS-TG containing biotinylated goat anti-mouse IgG (Vector, Burlingame, CA; 1:200) for 1.5 h at room temperature. Immunoreaction was visualized by incubation with ABC (one drop of reagents in 7 ml of 0.1 M PB; *ABC* Elite kits, Vector, Burlingame, CA), followed by diaminobenzidine histochemical reaction with 0.03% nickel ammonium sulfate.

## Visualization of CTB-gold

Sections were washed first with 0.1 M PBS, followed by 0.01 M PBS. An IntenSE M silver enhancement kit (Amersham plc, Amersham, UK) was used to visualize the CTB-gold signals (Sincich et al., 2007). A one-to-one cocktail of the IntenSE M kit solution and 33% gum Arabic solution was used as reagent. Development of reaction products was monitored under a microscope and terminated by rinsing the sections in 0.01 M PBS followed by several rinses in 0.1 M PBS. In general, the incubation time was approximately 2 h.

## Injection site determination

We determined the extent of injection site by the area in which the tracers filled the entire neuropil. In areas surrounding the injection site, the tracers labeled only cell somas, but not glial cells.

## Plotting of labeled neurons

The distribution of retrogradely labeled neurons was analyzed and plotted in sections with intervals of 500 μm. The specimens were analyzed under a Nikon Eclipse E-800 microscope (Nikon Co., Tokyo, Japan), at 4, 10, 20, and 100x resolutions. A microFIRE digital camera (MicroFire Technology Company Ltd., Shenzhen, China) was attached to the microscope to obtain digital data from the histological slides. With the digital section data thus obtained, the Neurolucida system (MBF Bioscience, Williston, VT, USA) was used for drawing the outer surface of the cortex, the borders between the gray and white matters and the middle of layer 4, and for plotting the labeled cells. The injection site where the entire neuropil was filled with the tracers was excluded from the labeled cell plotting. To determine the density of labeled neurons, we used a custom-made program (kindly gifted by Dr. Eiji Hoshi) on MATLAB (Mathworks, Natick, MA, USA) platform. The program enabled us to load and display the digitalized section data obtained from Neurolucida system to assign landmarks on the displayed sections and to align the positions of multiple sections on the basis of assigned landmarks. Using this program, we drew a curved line corresponding to layer 4 on each of the cortical sections, and labeled cells on each section were projected on to that line. The lines with projected neuronal densities were then unfolded and divided into 500 μm intervals. The number of labeled neurons within a square pixel of 500 μm by 500 μm (sections were plotted in every 500 μm) was taken as the density in that pixel. The density of labeled neurons in each pixel can be regarded as the density in a cortical column with a tangential area of 500 μm by 500 μm. The densities were then pseudo color-coded to make a cortical map of the density. We used the processed section data from the MATLAB program as inputs for the CARET package developed by the Van Essen laboratory (http://brainvis.wustl.edu/) and made flat maps of corresponding cortical surfaces. The pseudo color-coded density map obtained using the MATLAB program was then superimposed on the cortical flat map obtained from CARET to make a composite density-flat map of labeled neurons (**Figures 3, 5, 7**). All the flat maps and coronal section panels are presented as right hemispheres for the ease of comparison between the cases. The number of labeled neurons within each anatomical area (separately for superficial and deep layers) was divided by the number of labeled neurons in the area with the maximum number of labeled neurons (summed for superficial and deep layers) within each case to obtain normalized numbers (**Figure 9**). We also calculated a superficial layer vs. deep layer ratio of labeled neurons for the areas that consistently had labeled neurons in at least 2 cases (Table 1).

**Table 1:** **Total number of labeled neurons (N) and the ratio of the number of labeled neurons in superficial layers (layers 1–3) to the number of labeled neurons in deep layers (layers 5 and 6) (S/D) in each cortical area**.

**Area**	**Case 1L**		**Case 2R**		**Case 2L**	
	**N**	**S/D**	**N**	**S/D**	**N**	**S/D**
10m	5584	0.78	7713	1.13		
10o	587	0.69	33	0.83		
11m	1196	0.87	553	1.15		
11l	1200	1.33	49	2.77		
12r	1720	1.09				
12o	2794	1.62	556	0.99	13	0.63
12m	1398	2.26				
12l	1886	1.51	397	1.45	44	0.91
13b	604	0.84	45	0.88		
13l	514	1.68	692	0.88		
14r	1656	0.85				
46d	4432	1.03	510	0.71	50	3.55
46v	5019	0.76	465	0.73	132	0.45
45	1332	1.18	182	1.64		
9m	16778	2.32	14347	1.33	1249	1.49
9d	12375	1.27	3729	0.39	194	1.16
8Bm	9158	1.68	435	2.27	12771	5.15
8Bd	8514	1.56	118	0.93	1104	1.07
24a	1305	1.22	558	0.51	429	0.77
24b	2652	1.09	1660	1.28	998	1.11
24c	14807	1.4	5621	0.81	2482	1.21
23a	988	6.6	216	2.27	215	1.31
32	9939	1.27	2410	0.68	458	2.79
F6	231	1.33	308	1.88	10556	2.16
F7	578	1.31	56	0.81	3925	1.11
PrCo	537	1.03	50	0.14	32	0.38
Iai	1000	0.91	180	0.54	42	0.68
TAa	732	1.46	126	1.74		
TPO	1756	1.88	315	2.5	44	0.52
V2	944	5.51	13	1.17	25	0.19

*Labeled neurons were counted in 50-μm sections with 500 μm intervals*.

## Nomenclature

In this study, for nomenclature of cortical areas, we used combinations of area definitions of Brodmann (1905); Walker (1940); Petrides and Pandya (1999); Carmichael and Price (1995a,b); Preuss and Goldman-Rakic (1991) and Luppino et al. (1991). Area definitions of 8Bm and 9m in macaque monkeys were adapted from Walker (1940) and Petrides and Pandya (1999). To mark areas in coronal sections and flat maps, we used F99 flat map area definitions (Van Essen et al., 2011; Markov et al., 2014) and area definitions by Saleem and Logothetis (2007). The divisions of agranular frontal areas are adapted from the definitions of Matelli et al. (1991). F1 corresponds to area 4 of Brodmann (1905) and F3 corresponds to area 6 of Brodmann (1905) and Supplementary Motor Area (SMA) of Penfield and Welch (1951). F6 was defined as a part of area 6 of Brodmann (1905) and part of area 6aβ of Vogt and Vogt (1919). F7 was defined as a part of area 4 of Brodmann (1905) and a part of area 6aβ of Vogt and Vogt (1919). Luppino et al. (1993) defined F6 as pre-SMA, a separate functional area from SMA-proper (F3).

## Results

**Figure 2:**
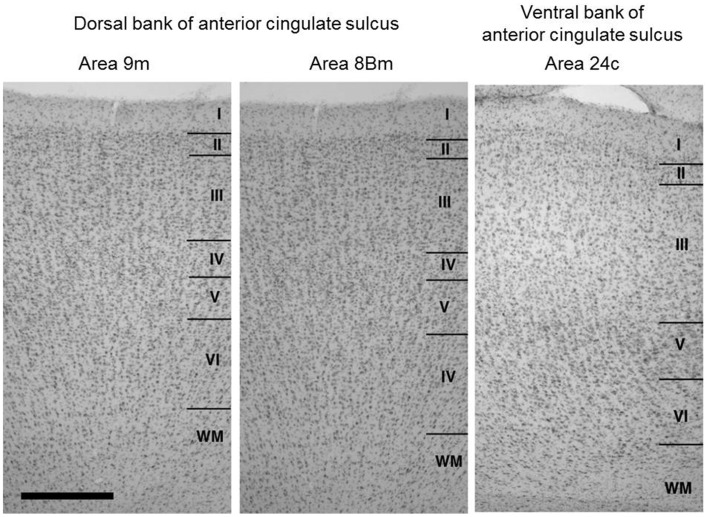
**Cytoarchitecture of the cortical areas around the injection sites (dorsal bank parts of areas 9m and 8Bm) together with that of the neighboring cortical area in the ventral bank of anterior cingulate sulcus (area 24c)**. Arabic numbers indicate layer numbers. Scale bar, 5 mm.

We identified layer 4, in Nissl stained sections, in the cortex extending over the dorsal bank of the anterior cingulate sulcus around the injection sites (Figure 2), whereas the ventral bank of the cingulate sulcus around the injection sites lacked layer 4. These indicate that the cortex extending over the dorsal bank of the anterior cingulate sulcus correspond to the ventral parts of areas 9m and 8Bm (Petrides and Pandya, 1999) and the cortex extending over the ventral bank of anterior cingulate sulcus corresponds to area 24c (Matelli et al., 1991; Arikuni et al., 1994). These findings were consistent with those in previous studies (Barbas and Pandya, 1989; Preuss and Goldman-Rakic, 1991; Petrides and Pandya, 1999).

The injection sites were located in the dorsal bank of the anterior cingulate sulcus in all the three cases (Figure 1), close to the dorsal lip of the sulcus in two cases (Case 1L and Case 2R) and at the middle between the dorsal lip and fundus in the third case (Case 2L). Based on anatomical landmarks, the injection site was judged to be located in area 9m in two cases (Case 1L and Case 2R) and in area 8Bm in the third case (Case 2L).

### Case 1L (Monkey 1, left hemisphere)

**Figure 3:**
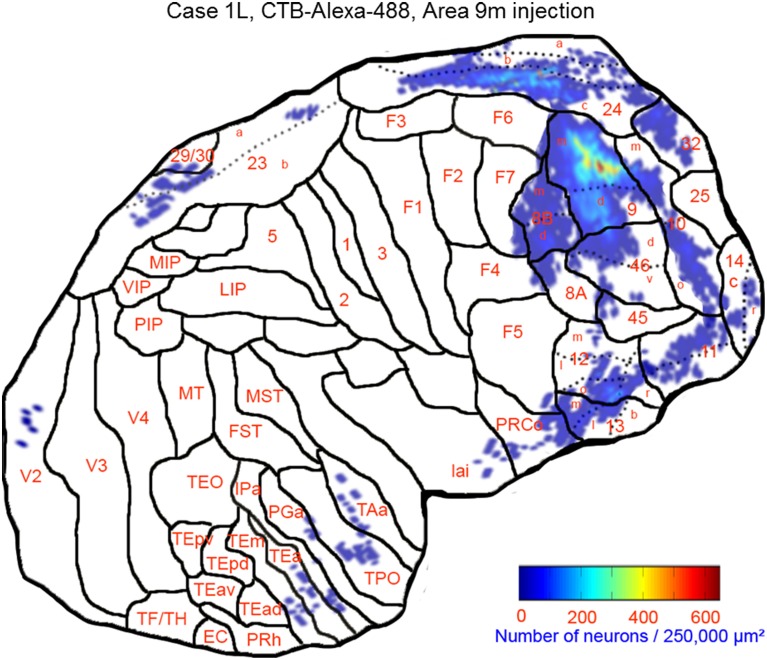
**Distribution of retrogradely labeled neurons on the flat map of the cerebral cortex in Case 1L**. The density of labeled neurons was converted into a pseudo-color-code. The flat map was flipped horizontally to make the comparison between the cases easier.

**Figure 4:**
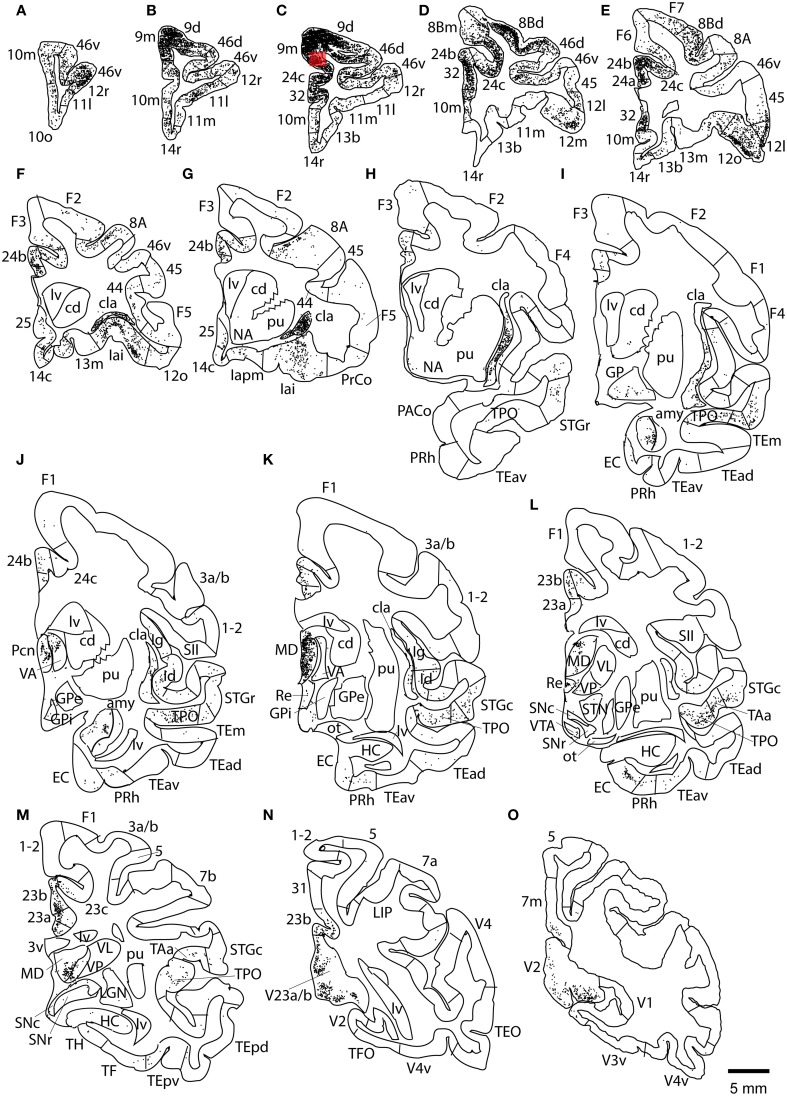
**Distribution of retrogradely labeled neurons on coronal sections in Case 1L (A–O)**. The abbreviations used to mark cortical areas are shown in the Abbreviations in the main text. The red square indicates the injection site. The coronal sections were flipped horizontally to make the comparison between the cases easier.

This case showed the most widespread cortical distribution of labeled neurons among the three cases, probably because the injection site was the largest among the three cases. The largest numbers of labeled neurons per area (>0.25 of the number of labeled neurons in the area with the maximum number of labeled neurons) were found in areas 9m, 9d, 8Bm, 8Bd, 46d, 46v, 10m, 24c, and 32. The labeled cells of intermediate numbers (between 0.10 and 0.25 of the maximum) occurred in areas 12r, 12l, 12o, 24b, and TPO. Smaller but definite numbers of labeled cells (between 0.01 and 0.10 of the number of labeled neurons in the area with the maximum number of labeled neurons) were observed in areas 10o, 11m, 11l, 12r, 12m, 13b, 13l, 14r, 45, 24a, 23a, F6, F7, PrCo, Iai, TAa, V23a/b, and V2 (Figures 3, 4A–O and Table 1). Among subcortical structures, dense distribution of labeled cells was found in the mediodorsal (MD), paracentral (Pcn), ventral anterior (VA) and reuniens (Re) nuclei of thalamus and claustrum (cla) (Figures 4F–L). There were less dense distributions of labeled cells in the basal nuclei of amygdala (amy) and in the hippocampus (HC) (Figures 4J–M). Labeled cells were also observed in the substantia nigra pars compacta (SNc) and ventral tegmental area (VTA) with denser distribution in SNc than in VTA (Figures 4L,M).

### Case 2R (Monkey 2, right hemisphere)

**Figure 5:**
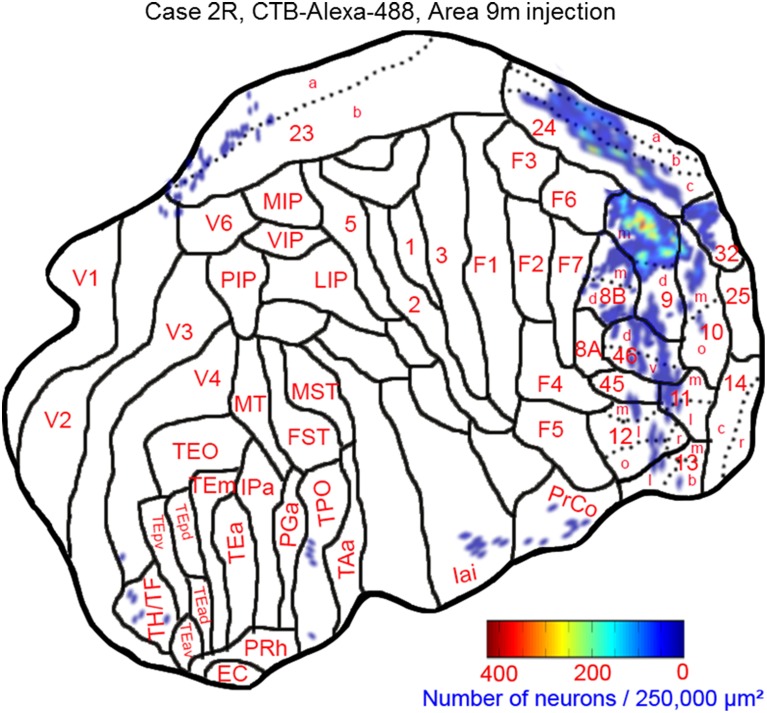
**Distribution of retrogradely labeled neurons on the flat map of the cerebral cortex in Case 2R**. The density of labeled neurons was converted into a pseudo-color-code.

**Figure 6:**
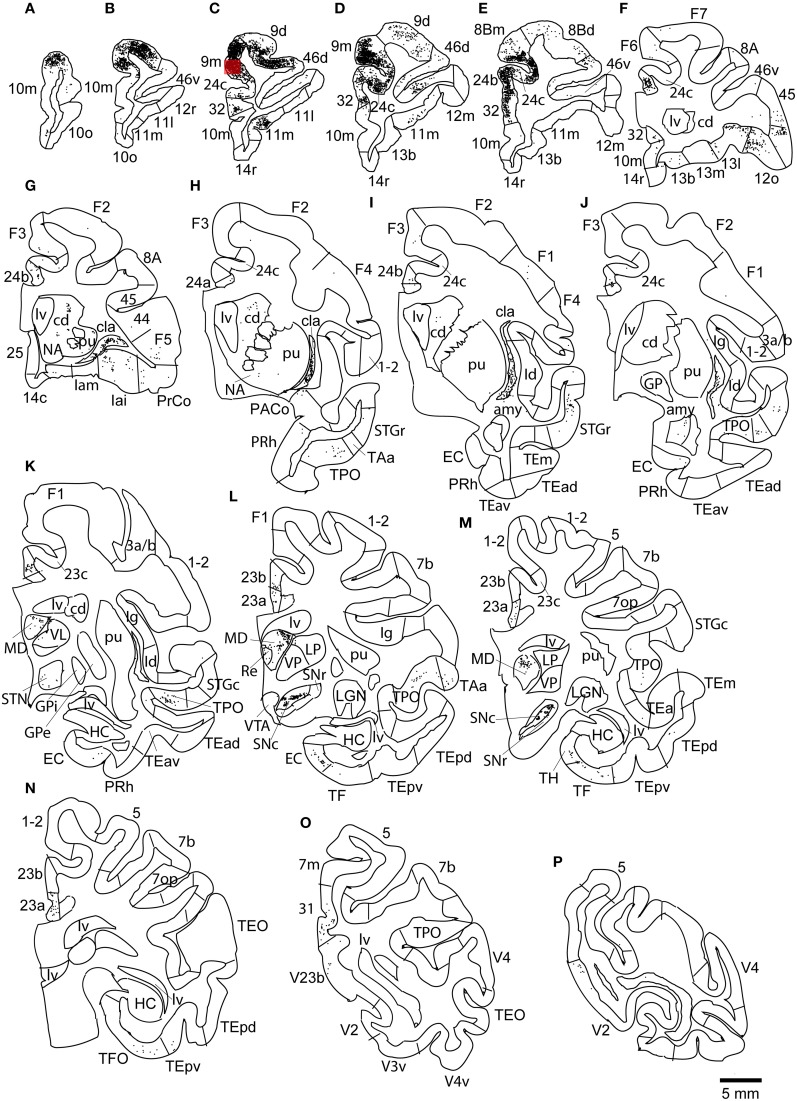
**Distribution of retrogradely labeled neurons on coronal sections in Case 2R (A–P)**. The abbreviations used to mark cortical areas are shown in the Abbreviations in the main text. The red square indicates the injection site.

The largest number of labeled cells were located in areas 9m, 9d, 10m, and 24c. Intermediate numbers of labeled cells were found in areas 24b and 32. Smaller numbers of labeled cells were observed in areas 11m, 12l, 12o, 13l, 45, 46d, 46v, 8Bm, 24a, 23a, F6, Iai, and TPO (Figures 5, 6A–P and Table 1). Among subcortical structures, dense distribution of labeled cells was observed in mediodorsal (MD) and reuniens (Re) nuclei of thalamus, substantia nigra pars compacta (SNc) and claustrum (cla) (Figures 6G–M). Less dense distributions of labeled cells were observed in the basal nuclei of amygdala (amy) and ventral tegmental area (VTA) (Figures 6J–M).

### Case 2L (Monkey 2, left hemisphere)

**Figure 7:**
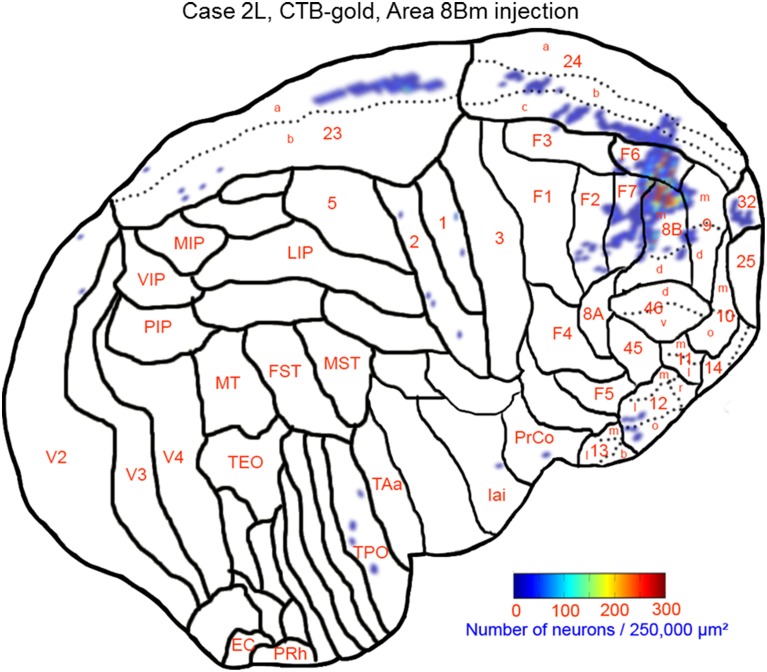
**Distribution of retrogradely labeled neurons on the flat map of the cerebral cortex in Case 2L**. The density of labeled neurons was converted into a pseudo-color-code. The flat map was flipped horizontally to make the comparison between the cases easier.

**Figure 8:**
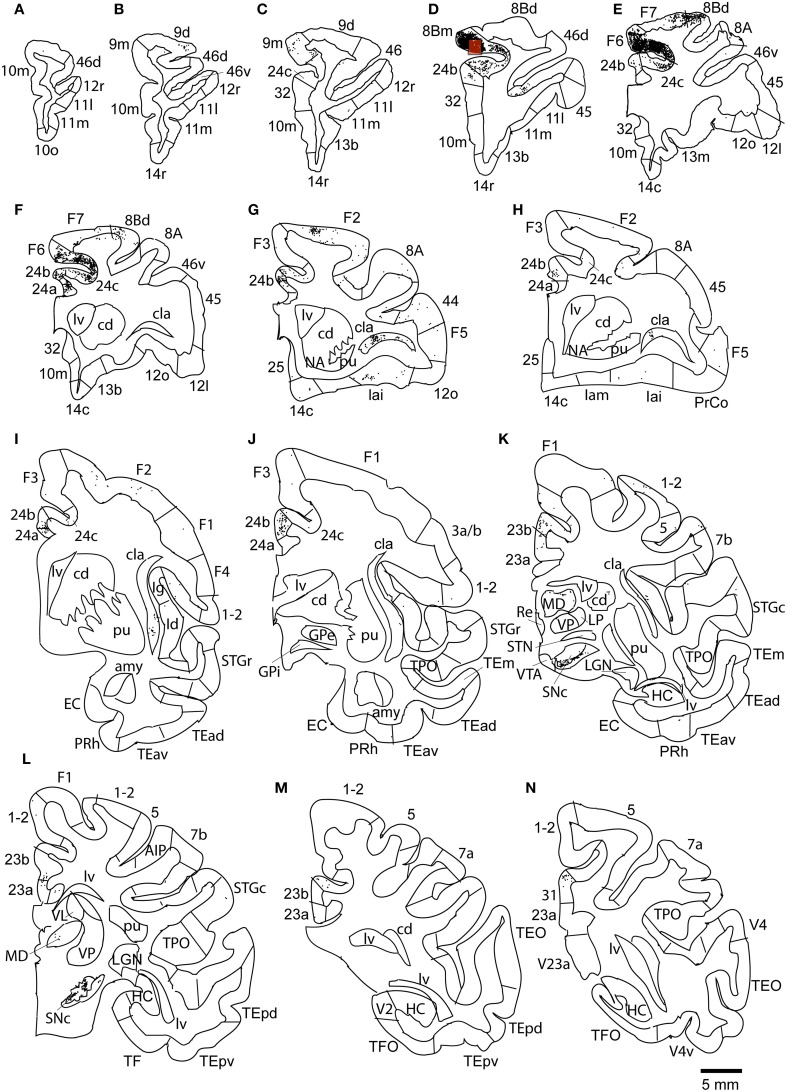
**Distribution of retrogradely labeled neurons on coronal sections in Case 2L (A–N)**. The abbreviations used to mark cortical areas are shown in the Abbreviations in the main text. The red square indicates the injection site. The coronal sections were flipped horizontally to make the comparison between the cases easier.

The largest number labeled cells were found area 8Bm, pre-supplementary motor area (F6) and dorsal premotor area (F7). Intermediate numbers of labeled cells were observed in area 24c. Smaller numbers of labeled cells were found in areas 46v, 9m, 9d, 8Bd, 32, 24a, 24b, 31, 23b, 23c, and 23a (Figures 7, 8A–N and Table 1). Sparse distribution of labeled cells was observed in the mediodorsal (MD) nucleus of thalamus, substantia nigra pars compacta (SNc) and claustrum (cla) (Figures 8K–L).

### Layer distribution of labeled cells

**Figure 9:**
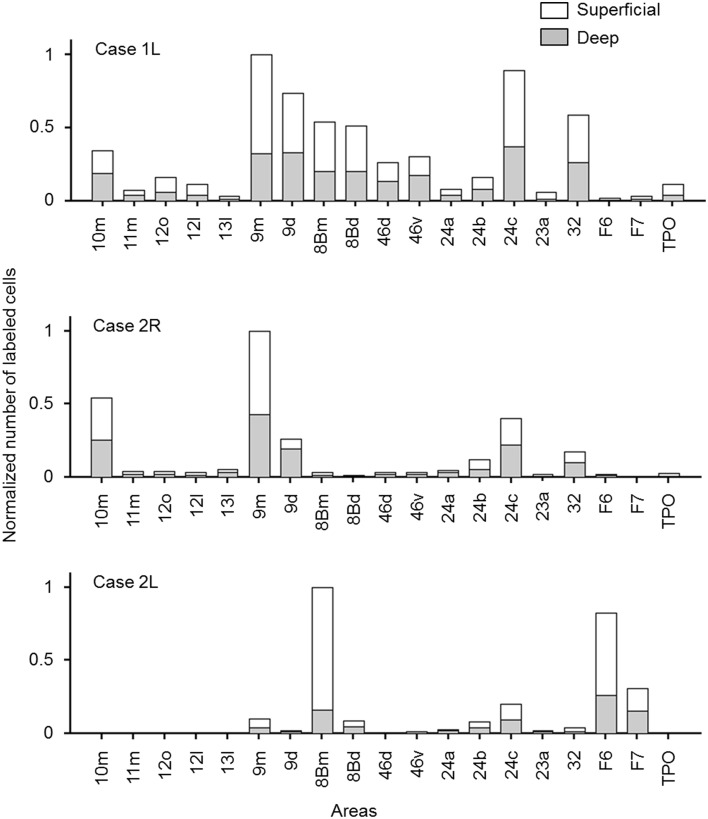
**Normalized numbers of retrogradely labeled neurons in each cortical area, counted separately for superficial and deep layers**. The number in each area was normalized by the total number of labeled neurons in the cortical area that had the maximum number of labeled neurons (a sum of the numbers in superficial and deep layers). Only the cortical areas in which labeled neurons were found in at least two of the three cases were included.

In most of the cortical areas where labeled cells occurred, the labeled cells were located in both the superficial layers (layers 1–3) and deep layers (layers 5 and 6). A large difference in the number of labeled cells between the superficial and deep layers with a ratio larger than 4 was found in a few cases, but the results were inconsistent between the cases (Figure 9 and Table 1).

## Discussion

In this study, we injected retrograde tracers (CTB-Alexa-488 or CTB-gold) into the sulcal portions of areas 9m and 8Bm from which we had previously recorded neuronal activities encoding particular action-outcome contingencies (Matsumoto et al., 2003), context novelty (Matsumoto et al., 2006), positive and negative prediction errors in values of executed actions (Matsumoto et al., 2007), and the history of erroneous responses (Kuwabara et al., 2014). The cortex extending over the dorsal bank of the anterior cingulate sulcus, where the injections were located, had layer 4 whereas the neighboring cortex extending over the ventral bank of the sulcus lacked layer 4. This indicated that the injection sites corresponded to the ventral parts of areas 9m and 8Bm (Petrides and Pandya, 1999). The distribution of labeled cells indicated that there were strong projections from the cingulate cortical areas (areas 24, 32, and 23) to the injection sites commonly in all the three cases. There were mutual connections between the sulcal parts of areas 9m and 8Bm. In addition, the sulcus part of area 9m received definite projections from other prefrontal areas (areas 46, 10, and 12) while the sulcus part of area 8Bm received minimal projections from these prefrontal areas. The sulcus part of area 8Bm, instead received strong projections from motor related areas (F6 i.e., the pre-supplementary motor area and F7 i.e., the dorsal premotor cortex) while the sulcus part of area 9m received minimal projections from these motor related areas. There were also common projections from superior temporal areas, only from area TPO or from surrounding areas as well. As for subcortical structures, there were common projections from the mediodorsal nucleus of thalamus, claustrum, and substantia nigra pars compacta. Projections from the ventral tegmental area were observed in two of the three cases, but the projections from the ventral tegmental area were weaker than those from the substantia nigra pars compacta. Minimal projection was observed in the basal nuclei of amygdala in the area 9m cases.

The distribution pattern of labeled cells after injections into the dorsal bank of the anterior cingulate sulcus in the present study was rather similar to those found after injections into the dorsal parts of areas 9 and 8B (areas 9d and 8Bd) (Barbas et al., 1999; Petrides and Pandya, 1999; Saleem et al., 2014). There was dense distribution of labeled cells in the dorsal surface of the prefrontal cortex, extending to area 46 in many cases, and also in the cingulate areas in common. In contrast, the distribution of labeled cells observed in our study was significantly different from those after injections into the ventral bank of anterior cingulate sulcus or the cingulate cortex below the anterior cingulate sulcus (Carmichael and Price, 1995a,b, 1996; Barbas et al., 1999) in that the distribution of labeled cells on the dorsal surface of prefrontal cortex (areas 9d and 8Bd) was clear in our cases while sparse or absent in the latter cases with ventral injections.

The rather clear difference between the projections to the sulcus part of area 9m and those to the suclus part of area 8Bm (projections from areas 9d, 10 and 12 to the sulcus part of area 9m vs. projections from pre-motor areas to the sulcus part of area 8Bm) was unexpected because we have not experienced a clear discontinuity along the rostral-caudal axis in neuronal properties recorded from the dorsal bank of the anterior cingulate sulcus (Matsumoto et al., 2003, 2006, 2007; Kuwabara et al., 2014). The mutual connections between the two areas in the dorsal bank of the anterior cingulate sulcus may be strong enough to convey motor-related information from area 8Bm to area 9m and cognitive-control-related information from area 9m to area 8Bm. Alternatively, observations with new behavioral paradigms may find differences in neuronal properties between the two areas. However, because we had only one case with tracer injection into area 8Bm, further studies are necessary to definitely conclude the difference between the projections to the sulcus part of area 9m and those to the sulcus part of area 8Bm.

A general principle has been proposed for cortico-cortial projections within the prefrontal cortex regarding layer localization of cells-of-origin and terminals: projections from an agranular area to a granular area originate in deep layers and terminate in superficial layers while projections from a granular area to an agranular area originate in superficial layers and terminate in deep layers (Barbas and Hilgetag, 2002; Barbas and Zikopoulos, 2007). The projections in our cases, i.e., those from various prefrontal, cingulate and pre-motor areas to the cingulate sulcus parts of areas 9m and 8Bm didn't take either type in the layer distribution of cells-of-origin, which are consistent with the proposal as layer 4, though present, was not fully distinguished in areas 9m and 8Bm as compared with that in the lateral prefrontal areas.

The present study showed subcortical distribution of labeled neurons in the claustrum (cla), mediodorsal nucleus of thalamus (MD), substantia nigra pars compacta (SNc), consistently across the cases. Projections from the claustrum to prefrontal cortical areas have been found to be widespread (Tanne-Gariepy et al., 2002). Differential spatial distributions of labeled neurons in the claustrum have been observed after retrograde tracer injections into different motor cortical areas (Tanne-Gariepy et al., 2002) and the distribution of labeled neurons was limited to a rostral part of the claustrum after retrograde tracer injections into the dorsal aspect of area 9 (area 9d) (Reser et al., 2014). However, in the present study, we didn't observe any spatial segregation of labeled neurons in the claustrum following retrograde injections into area 9m and area 8Bm. The labeled axon terminals had previously been found widely in the prefrontal, frontal and cingulate cortical areas after retrograde tracer injections to the substantia nigra pars compacta (SNc) (Haber et al., 2000). Layer specific distribution of dopamine receptors have been observed in prefrontal areas of macaque monkeys including cortical areas on the dorsal bank of cingulate sulcus (Goldman-Rakic et al., 1990) with relatively high density in dorsomedial region, area 9 (Lewis, 1992; Akil et al., 1999).

The neurons projecting from the substantia nigra pars compacta and ventral tegmental area to the dorsal bank of the anterior cingulate sulcus may be dopaminergic. The dopaminergic afferents may convey the information of prediction errors in action values (Schultz et al., 1997) to the dorsal bank of the anterior cingulate sulcus (Holroyd and Coles, 2002). Many cells in this cortical area respond to action outcomes only during learning phase in which outcomes are not well predicted (Matsumoto et al., 2007; Quilodran et al., 2008; Kuwabara et al., 2014). Different groups of cells in the dorsal bank of the anterior cingulate sulcus increase their firing rates to negative and positive prediction errors, whereas dopamine cells increase and decrease their firing rates to positive and negative prediction errors, respectively (Schultz et al., 1997). It is not known where the reversal in response direction to negative outcomes occurs. There are also cells in the dorsal bank of the anterior cingulate sulcus that increase their firing rates to both positive and negative prediction errors (Matsumoto et al., 2007). The afferents from the cingulate areas may convey this signal of unsigned errors. A recent study of simultaneous single-cell recordings from the basal nuclei of amygdala and areas 24c and 24b showed that the signal of unsigned errors in classical conditioning paradigm first occur in the amygdala followed by that in areas 24c and 24b (Klavir et al., 2013). The projections from areas 24c and 24b may provide the dorsal bank of the anterior cingulate sulcus the signal of unsigned errors. The afferents from the mediodorsal nucleus of thalamus may also convey the information of negative outcomes. The error positivity signals in electrocorticograms disappeared after lesions of the mediodorsal nucleus (Seifert et al., 2011; Ullsperger et al., 2014).

Afferents from the premotor areas (the dorsal premotor cortex and pre-supplementary motor area) may convey information of actions during learning and possibly during execution. Lesions of the anterior cingulate sulcus damaged the value-based action selection when the action-outcome contingency was unstable (Kennerley et al., 2006; Rudebeck et al., 2008). Activities of cells in the dorsal bank of the anterior cingulate sulcus represented action sequences (Procyk et al., 2000), particular action-outcome contingency (Matsumoto et al., 2003) and directions of intended actions (Cai and Padoa-Schioppa, 2012). Afferents from areas 9d, 46, and 10 may convey the information of general task condition and context (Duncan and Owen, 2000; Miller and Cohen, 2001; Paus, 2001).

The diverse inputs related to different aspects of outcomes along with the motor related inputs from the premotor areas and the context information from the dorsal and dorsolateral prefrontal areas, may make the dorsal bank of the anterior cingulate sulcus an optimal anatomical location to integrate goal directed action plans with various types of value and context information.

## Author contributions

HA, MM, KM, and NI made tracer injections. MKE and NI observed the sections and analyzed the data. MKE, KT, and NI wrote the manuscript.

### Conflict of interest statement

The authors declare that the research was conducted in the absence of any commercial or financial relationships that could be construed as a potential conflict of interest.

## Abbreviations

10m, Area 10: medial subdivision
10o, Area 10: orbital subdivision
11l, Area 11: lateral subdivision
11m, Area 11: medial subdivision
1–2: Somatosensory areas 1 and 2
12l, Area 12: lateral subdivision
12m, Area 12: medial subdivision
12o, Area 12: orbital subdivision
12r, Area 12: rostral subdivision
13b, Area 13: subdivision on the medial bank of olfactory sulcus
13l, Area 13: subdivision on the lateral part of middle orbital gyrus
14c: Area 14 caudal subdivision
14r, Area 14: rostral subdivision
23a, Area 23: ventral subdivision
23b, Area 23: dorsal subdivision
24a, Area 24: ventral subdivision near callosal sulcus
24b, Area 24: central subdivision
24c, Area 24: dorsal subdivision in ventral bank of anterior cingulate sulcus
29: Area 29
3: Somatosensory area 3
30: Area 30
31: Area 31 of posterior cingulate gyrus
32: Area 32
3a/b: Somatosensory areas 3a and 3b
45: Area 45
46d, Area 46: dorsal subdivision
46v, Area 46: ventral subdivision
7b: Visual area 7b
7op: area 7op (parietal operculum)
8A: Area 8A
8Bd, Area 8B: dorsal subdivision
8Bm, Area 8B: medial subdivision
9d, Area 9: dorsal subdivision
9m, Area 9: medial subdivision
AIP: Anterior intraparietal area
amy: Amygdala
cd: Caudate nucleus
cla: Claustrum
EC: Entorhinal cortex
F1: Agranular frontal area F1
F2: Agranular frontal area F2
F3: Agranuar frontal area F3
F4: Agranular frontal area F4
F5: Agranular frontal area F5
F6: Agranular frontal area F6
F7: Agranular frontal area F7
FST: Area FST in the sts floor
GP: Globus pallidus
GPe, Globus pallidus: external segment
GPi, Globus pallidus: internal segment
HC: Hippocampus
Iai: Intermediate agranular inuslar area
Iam: Medial agranular insular area
Iapm: Posteromedial agranular insula
Id: Dysgranular insula
Ig: Granular insula
IPa: Area IPa in the sts fundus
LGN: Lateral geniculate nucleus
LIP: Lateral Intraparietal area
LP: Lateral posterior nucleus
lv: Lateral ventricle
MD: Medial dorsal nucleus of thalamus
MIP: Medial Intraparietal area
MST: Medial superior temporal area (visual)
MT: Middle temporal area (visual)
NA: Nucleus accumbens
ot: Optic tract
PACo: Periamygdaloid cortex o
Pcn: Paracentral nucleus of thalamus
PGa: Area PGa
PIP: Posterior Intraparietal area
PrCo: Precentral opercular area
PRh: Perirhinal cortex
pu: Putamen
Re: Reuniens nucleus of thalamus
SII: Secondary somato sensory area
SN: Substantia nigra
SNc: Substantia nigra pars compacta
SNr: Substantia nigra pars reticulate
STN: Subthalamic nucleus
sts: Superior temporal sulcus
TAa: Area TAa in the sts dorsal bank (auditory)
TEad: Dorsal subregion of anterior TE (visual)
TEav: Ventral subregion of anterior TE (visual)
TEm: Area TEm in the sts ventral bank (visual)
TEO: Area TEO (visual)
TEpd: Dorsal subregion of posterior TE (visual)
TEpv: Ventral subregion of posterior TE (visual)
TF/TH: Area TF and TH of parahippocampal cortex
TFO: Area TFO of parahippocampal cortex
TPO: Area TPO (polysensory)
V1: Visual area V1
V2: Visual area V2
V23a/b: area V23a and V23b in posterior cingulate cortex
V3: Visual area V3
V3v, Visual area 3: ventral part
V4: Visual area V4
V4v, Visual area 4: ventral part
VA: Ventral anterior nucleus
VIP: Ventral Intraparietal area
VL: Ventral lateral nucleus
VP: Ventral posterior nucleus
VTA: Ventral Tegmental Area.
